# Discovery and characterization of novel jeilongviruses in wild rodents from Hubei, China

**DOI:** 10.1186/s12985-024-02417-8

**Published:** 2024-06-25

**Authors:** Min Gan, Bing Hu, Qingwen Ding, Nailou Zhang, Jinbo Wei, Tao Nie, Kun Cai, Zhenhua Zheng

**Affiliations:** 1 Key Laboratory of Special Pathogens and Biosafety, Wuhan Institute of Virology, Center for Biosafety Mega-Science, Chinese Academy of Sciences, Wuhan, 430071 Hubei China; 2 University of Chinese Academy of Sciences, Beijing, 100049 People’s Republic of China; 3 Hubei Provincial Center for Disease Control and Prevention, Wuhan, 430079 Hubei China; 4 Xianning Municipal Center for Disease Control and Prevention, Xianning, 437199 Hubei China

**Keywords:** Jeilongvirus, Rodent, Paramyxovirus, PCR screening, Prevalence

## Abstract

The genus *Jeilongvirus* comprises non-segmented negative-stranded RNA viruses that are classified within the *Paramyxoviridae* family by phylogeny. Jeilongviruses are found in various reservoirs, including rodents and bats. Rodents are typical viral reservoirs with diverse spectra and zoonotic potential. Little is currently known about jeilongviruses in rodents from central China. The study utilized high-throughput and Sanger sequencing to obtain jeilongvirus genomes, including those of two novel strains (HBJZ120/CHN/2021 (17,468 nt) and HBJZ157/CHN/2021 (19,143 nt)) and three known viruses (HBXN18/CHN/2021 (19,212 nt), HBJZ10/CHN/2021 (19,700 nt), HBJM106/CHN/2021 (18,871 nt)), which were characterized by genome structure, identity matrix, and phylogenetic analysis. Jeilongviruses were classified into three subclades based on their topology, phylogeny, and hosts. Based on the amino acid sequence identities and phylogenetic analysis of the L protein, HBJZ120/CHN/2021 and HBJZ157/CHN/2021 were found to be strains rather than novel species. Additionally, according to specific polymerase chain reaction screening, the positive percentage of Beilong virus in Hubei was 6.38%, suggesting that Beilong virus, belonging to the *Jeilongvirus* genus, is likely to be widespread in wild rodents. The identification of novel strains further elucidated the genomic diversity of jeilongviruses. Additionally, the prevalence of jeilongviruses in Hubei, China, was profiled, establishing a foundation for the surveillance and early warning of emerging paramyxoviruses.

**Supplementary Information:**

The online version contains supplementary material available at 10.1186/s12985-024-02417-8.

## Highlights

1. Two novel jeilongvirus strains (HBJZ157/CHN/2021 and HBJZ120/CHN/2021) were discovered and characterized.

2. The prevalence of jeilongviruses in rodents from central China was investigated, revealing that the Beilong virus is widespread.

**Supplementary Information:**

The online version contains supplementary material available at 10.1186/s12985-024-02417-8.

## Introduction

Over the past two decades, emerging infectious diseases have caused continuous public health emergencies. Epidemiological investigations and viral evolutionary studies have shown that the pathogens responsible for these diseases mainly originate from wild animals, including bats [1], rodents [2], birds [3], and other reservoirs [4] that harbor a diverse spectrum of viruses with zoonotic potential. Rodents, which are distributed worldwide and comprise over 2,200 taxa, occupy a variety of habitats, including wild biotopes, artificial surroundings, and areas between wild and human communities [5, 6]. As a result, rodents serve as links between humans, domestic animals, and wildlife. Numerous epidemiological investigations have shown that certain members of *Paramyxoviridae*, *Hantaviridae*, *Arenaviridae*, *Picornaviridae*, and other viral pathogen families carried by rodents pose a potential threat to humans and livestock [7–11].

The family *Paramyxoviridae* is a group of non-segmented negative-stranded RNA viruses coated with envelopes that possess morphologically pleomorphic features. They are classified into 9 subfamilies and 23 genera [12, 13], including *Henipavirus*, *Morbillivirus*, *Respirovirus*, *Orthoavulavirus*, and *Orthorubulavirus*, which are significant pathogens that cause diseases in humans and animals [14]. Many novel paramyxoviruses have been detected and/or isolated from samples related to rodents in recent decades, including henipaviruses [15], jeilongviruses [2, 16–22], and narmoviruses [21, 23–25]. The genus *Jeilongvirus*, initially proposed in 2005, includes 32 species recognized by the International Committee on Taxonomy of Viruses (ICTV), including *Jeilongvirus queenslandense* (J virus, JV), *Jeilongvirus beilongi* (Beilong virus, BeiV), and *Jeilongvirus tailamense* (Tailam virus, TaiV [12]). Molecular epidemiological investigations have shown that jeilongviruses are hosted by rodents, bats, hedgehogs, and other animals [2, 26–29]. Equally important is research on infection models of JV strains, which further reveals their pathogenicity in mice [30]. Furthermore, the distribution of multiple known or novel jeilongviruses discovered in several regions of China (Inner Mongolia, Xinjiang, Northeast, Southwest, North, South, and Central China) exhibits host and regional specificity [31–33]. However, due to insufficient systematic research on a significant number of rodent-related samples, little is known about the prevalence and phylogenetic characteristics of jeilongviruses in rodents from central China.

In this study, we identified five species in the genus *Jeilongvirus* from a large number of rodent samples collected using a bioinformatics workflow and polymerase chain reaction (PCR) screening. We characterized two novel jeilongviruses based on genome structure, identity matrix, and phylogenetic analysis. Through profiling the prevalence of jeilongviruses in Hubei, China, we established a foundation for further research on the surveillance and early warning of paramyxoviruses.

## Materials and methods

### Sample collection

**Fig. 1:**
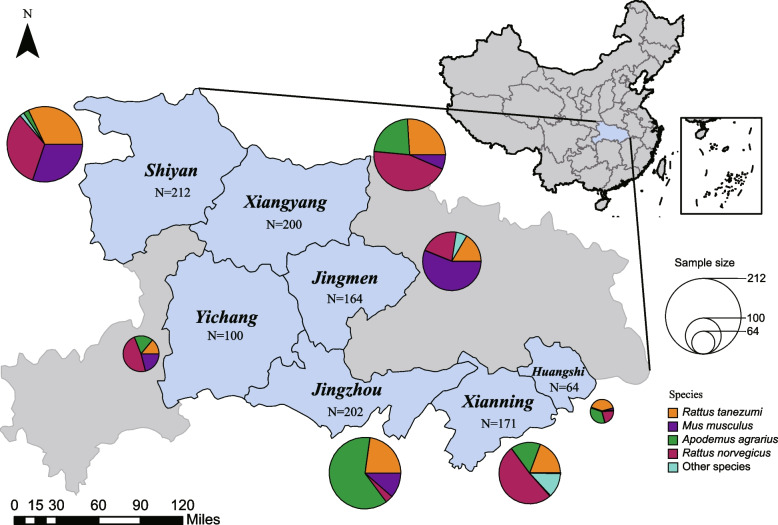
Sampling sites in this study. Seven prefecture-level cities in Hubei sampled are highlighted in blue. The circle adjacent to each city illustrates the size (the size of circles) and species (the color of pies) of samples

Field sampling was conducted from March to December 2021 in Hubei, a province in central China, at sites in seven prefecture-level cities: Xianning, Shiyan, Xiangyang, Jingmen, Jingzhou, Huangshi, and Yichang (Fig. 1). The sampling sites included various habitats, such as forests, fields, caves, villages, and urban areas. Small mammals, including rodents and shrews, were either trapped alive using traps or mortally using rat clips. The traps were set near mouse holes at dusk and checked the following morning. We collected 1113 individuals belonging to eight rodent species from four genera across all cities: Xianning (*n* = 171), Shiyan (*n* = 212), Xiangyang (*n* = 200), Jingmen (*n* = 164), Jingzhou (*n* = 202), Huangshi (*n* = 64), and Yichang (*n* = 100). These animals were identified by experienced field biologists based on morphology, and tissue specimens, including the heart, liver, spleen, lung, and kidney, were collected and preserved in *RNAlater* or viral transport medium. The sampling tubes were immediately packed according to their source and covered with dry ice to maintain a low storage temperature. The samples were subsequently transferred to -80 ℃ refrigerators in the laboratory.

### DNA/RNA extraction

Samples immersed in *RNAlater* were homogenized using a tissue cell destroyer DS1000 (NZK Biotech, Wuhan, China) under a shearing force provided by grinding beads with a diameter of 3 mm. The supernatant of the tissue homogenate was collected and combined with other tissues in advance (a subset composed of ten tissue samples), followed by DNA/RNA extraction using an Automatic Nucleic Acid Extractor VNP-32P equipped with a Virus DNA/RNA Extraction Kit (Vazyme Biotech, Nanjing, China), according to the manufacturer's instructions. To obtain high-quality concentrated RNA for partial genomic amplification and rapid amplification of cDNA ends, the supernatant was treated with the FastPure® Viral DNA/RNA Mini Kit (Vazyme) following the manufacturer's recommendation: 200 μl of the conflated supernatant was mixed with 500 μl Buffer VL for lysis then transferred to FastPure® RNA Columns and centrifuged at 12,000 revolutions per minute. The filtrate was discarded. Then, 600 μl of Buffer RW was added, and the filtrate was removed to obtain a pure final product. Finally, RNase-free ddH_2_O was used to elute RNA from columns, which was stored at -80 ℃. For next-generation sequencing, the supernatant was subjected to RNA extraction using the QIAamp® Viral RNA Mini Kit (Qiagen, Hilden, Germany) according to the manufacturer's instructions. All procedures were carried out in a sterile and nuclease-free environment to avoid cross-contamination and degradation.

### Rodent identification

Each sample morphologically identified in this study was confirmed using a conserved DNA barcode. PCR was performed to amplify a partial mitochondrial cytochrome b gene (CytB) based on DNA extracted from tissues using the primer set developed by Schlegel and colleagues (Table 1) [34]. The purified amplicon was sent to Sangon Biotech (Shanghai, China) for Sanger sequencing. Sequencing results were assembled into integrated sequences with SeqMan (V7.1.0) and identified by manipulating the Nucleotide Basic Local Alignment Search Tool (BLASTn, https://blast.ncbi.nlm.nih.gov/Blast.cgi) against the database nucleotide collection (nr/nt).

**Table 1:** Primers of identification and screening used in the study

Primers	Sequences
**Host identification**	
CytB-F	5′-TCATCMTGATGAAAYTTYGG-3′
CytB-R	5′-ACTGGYTGDCCBCCRATTCA-3′
**RT-PCR screening**	
BeiV-N-F-200	5′-AGCTGATCTTGCTGTGAGAA-3′
BeiV-N-R-200	5′-AGGATAGTCCCTAGATCAGC-3′
BeiV-P-F-450	5′-GATCATGAGATGTCTGCTGC-3′
BeiV-P-R-450	5′-CCGGTAGATAGTCTAGCTTC-3′
BeiV-G-F-150	5′-CCTTGCCATGTCGAATTTCT-3′
BeiV-G-R-150	5′-GTCACATTGAGGGCAATGAT-3′
BeiV-L-F-300	5′-GGTGGCTATGATTGAACCTC-3′
BeiV-L-R-300	5′-AGGTTTGTTCATATGTGCTC-3′
JMAaJV-1-N-F-150	5′-GTCAGCAAGATCATCAGGAGC-3′
JMAaJV-1-N-R-150	5′-TCTTGTAGCAAGTTTGATGTTGTC-3′
JMAaJV-1-P-F-300	5′-GAGCTTATACAACAGAATCCGGAG-3′
JMAaJV-1-P-R-300	5′-CTCTGCTACTGTTCCCATCAC-3′
JMAaJV-2-G-F-150	5′-GCCTATGCACATGCTATAACAC-3′
JMAaJV-2-G-R-150	5′-CAGTGACAGAACATGATCTCCTG-3′
JMAaJV-2-L-F-350	5′-CCACACTGTCCACCATATAACAC-3′
JMAaJV-2-L-R-350	5′-CTTCAGGTTATGTCCGATCCC-3′
nParaV1-N-F-150	5′-GCCCTACTGGTTACAGTGATGA-3′
nParaV1-N-R-150	5′-CATCAGGAGCATTGTCCGGA-3′
nParaV1-P-F-350	5′-TTGCTGCTTCTGGGAGTGAT-3′
nParaV1-P-R-350	5′-TTAGACTGCCAAGTTCTTCACC-3′
nParaV1-G-F-250	5′-ACCAAGTGATAAGGAAGAAGCTG-3′
nParaV1-G-R-250	5′-CACCATCAAGCATAGACCTGG-3′
nParaV2-L-F1-350	5′-TGAAACAATAGTCAGCTCGC-3′
nParaV2-L-R1-350	5′-AATTAAATCCACCCAATTGTGAGG-3′
nParaV2-L-F2-200	5′-CAATAGTCAGCTCGCATTTCTT-3′
nParaV2-L-R2-200	5′-CAAATGCTATAACCTAACCACCTAC-3′

### Multiple RT-PCR and nested PCR screening

RNA was automatically extracted from the conflated supernatant and reverse-transcribed (RT) using FastKing gDNA Dispelling RT SuperMix (Tiangen Biotech, Beijing, China). Each 20 μl reaction system consisted of 4 μl of 5 × RT Mix, 3 μl of RNA, and 13 μl of ddH_2_O. The reaction program followed the manufacturer's instructions: incubation at 42 ℃ for 15 min, followed by enzyme inactivation at 95 ℃ for 3 min. The resulting cDNA was screened using multiple PCR (for BeiV, Jingmen Apodemus agrarius Jeilongvirus 1 (JMAaJV-1), Jingmen Apodemus agrarius Jeilongvirus 2 (JMAaJV-2), and HBJZ120/CHN/2021) and nested PCR (for HBJZ157/CHN/2021) using primer sets based on intraspecifically conserved protein-coding sequences (Table 1). Each 25 μl reaction system comprised 12.5 μl of 2 × Rapid Taq Master Mix (Vazyme), 2 μl of 10 μM primer sets (with the proportions of several sets distributed equally in volume), 1 μl of cDNA, and 9.5 μl of ddH_2_O. The reaction was carried out manipulating Applied Biosystems (Thermo Fisher Scientific, China) with the following program: pre-denaturation at 95 ℃ for 5 min, followed by 40 cycles of denaturation at 95 ℃ for 15 s, annealing at the melting temperature (T_m_) of the primer sets for 15 s, extension at 72 ℃ for 5 s, and thorough extension at 72 ℃ for 5 min. The nested PCR (NPCR) consisted of outer and inner PCR. The outer PCR aimed to amplify longer target sequences, and the inner PCR was performed to obtain shorter target sequences. To confirm the presence of positive samples, the PCR products were analyzed by agarose gel electrophoresis. The primer sets based on diverse sequences yielded ladder-like graphs of putatively positive products. Positive samples were confirmed using Sanger sequencing.

### Genomic sequencing

Specific extracted RNA was pooled for next-generation sequencing using the MGI2000 100-bp or 150-bp paired-end (PE100 or PE150) platform (BGI, China). Details of the pools and read lengths are listed in Table S1. Raw data were filtered to remove low-quality reads and adapters employing fastp (https://github.com/OpenGene/fastp). Subsequently, clean data were assembled into contigs de novo using the MEGAHIT software. All assembled jeilongvirus-related contigs were confirmed using BLAST. First, BLASTn was manipulated as described above. Then, the Translated Basic Local Alignment Search Tool (BLASTX, https://blast.ncbi.nlm.nih.gov/Blast.cgi) was utilized against the non-redundant protein sequences (nr) database using the standard genetic code. Based on the selected jeilongvirus-related contigs, specific primers (Table S2) were designed to amplify multiple fragments whose 5’ and 3’ termini overlapped partially by Phanta® Max Super-Fidelity DNA Polymerase (Vazyme). The resulting amplicons were subjected to Sanger sequencing to correct for assembled jeilongvirus-related sequences.

### Rapid amplification of cDNA ends

Several gene-specific primers and nested gene-specific primers (Table S2) were designed to further complete the genomes of jeilongviruses based on the assembled and corrected sequences as described above. The rapid amplification of cDNA ends (RACE) was performed using the HiScript-TS 5'/3' RACE Kit (Vazyme) following the manufacturer's instructions: first-strand cDNA was synthesized with random primers and diluted to generate RACE-ready cDNA, and 5'/3' terminal products were amplified by PCR/NPCR and purified using the FastPure® Gel DNA Extraction Mini Kit (Vazyme). The produced 5'/3' terminal sequences were cloned using the pGEM T-Easy vector system (Promega, Madison, WI, USA) and determined by Sanger sequencing.

### Phylogenetic and genomic analysis

The relevant virus sequences were obtained from NCBI Virus (www.ncbi.nlm.nih.gov/labs/virus/vssi/), followed by the substitution of degenerate bases with seqkit [35] v2.4.0. Alignments of separate ORFs were performed using MAFFT [36] v7.490, and the eight major ORFs (N, P, M, F, SH, TM, G, and L) of each genome were concatenated for alignment in the same manner. Subsequently, misaligned sequences were trimmed using TrimAl v1.4.1, and repetitive sequences were deleted using Seqkit. To search for the best models of maximum likelihood (ML) trees, modeltest-ng [37] v0.1.7 was used to assess diverse models. ML phylogenetic trees were then inferred in iqtree [38] v2.2.0_beta based on nucleotide (for concatenated primary ORFs) or amino acid sequences (for N, P, M, F, RBP, and L proteins) using appropriate models and visualized using iTOL [39] (https://itol.embl.de/). All ORFs were identified using the ORF prediction tool (ORF Finder, https://www.ncbi.nlm.nih.gov/orffinder/) with coding sequencing (CDS) information obtained from GenBank. The genome organization of jeilongviruses drawn from gggenomes v0.9.5.9000 was integrated into an ML tree based on concatenated primary ORFs. The identity matrix at the amino acid level was constructed as follows: the concatenated major ORFs (N, P, M, F, SH, TM, G, and L) and the N, P, M, F, G, and L ORFs were aligned separately using MAFFT. Pairwise similarity analysis was performed using the default parameters in BioAider v1.423 [40], and Excel v2211 was used to visualize the heat maps.

## Results

### Genomic characteristics of novel jeilongvirus strains

Based on high-throughput sequencing (HTS) of 29 pooled RNA libraries and bioinformatics analysis, approximately 2.8 billion reads were obtained. The number of reads generated by each pool is detailed in Table S1. Approximately 3.45 million paramyxovirus reads were obtained, accounting for approximately 0.123% of the total reads. Thirty jeilongvirus-related contigs were assembled and annotated. Nearly complete genomes of three members of the genus *Jeilongvirus* were identified: HBXN18/CHN/2021 (19,212 nt, complete), HBJZ10/CHN/2021 (19,700 nt, lack of two termini), HBJM106/CHN/2021 (18,871 nt, lack of termini) as BeiV, JMAaJV-1, and JMAaJV-2, respectively. In addition to these known virus species, two other jeilongvirus-related genomes were identified using a combination of Sanger sequencing for gap completion and BLASTn analysis. One of these genomes, named HBJZ157/CHN/2021 (19,143 nt, lack of termini), appeared to be the most similar to Rattus tanezumi Jeilongvirus (accession number: OR233793), sharing 98.52% nucleotide identity. The other genome, named HBJZ120/CHN/2021 (17,468 nt, lack of termini), shared only 89.41% nucleotide identity with Paju Apodemus paramyxovirus 2 (PAPV-2, accession numbers: MT823463 and MT823464).

**Fig. 2:**
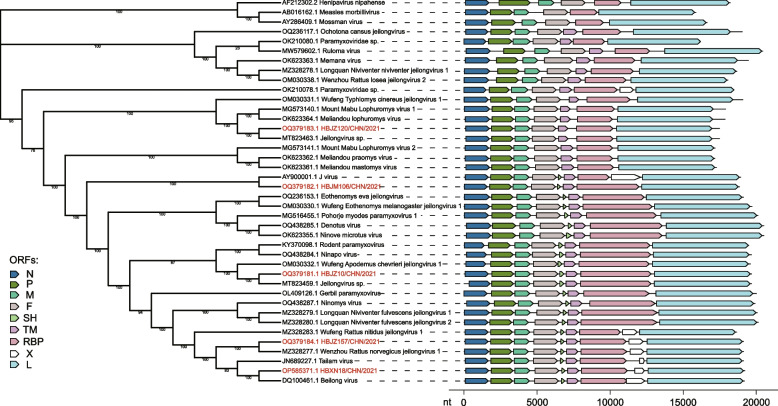
Phylogenetic tree and genome organization of jeilongviruses. Strains identified in this study are highlighted in red. Virus and strain names on leaves are listed in Table S4. The tree was constructed by using a ML method based on nucleotide sequences of concatenated primary ORFs. Rectangle arrow colors indicate major ORFs according to the legend, the genome organization on the right was drawn in accordance with the scale at the bottom

Using ORF prediction tools, the gene start, stop, and intergenic regions (IGR) of the novel jeilongvirus-related strains were found to be highly conserved and are listed in Table S3. Similar to the majority of species in the genus *Jeilongvirus*, the genomes of JMAaJV-1, JMAaJV-2, and HBJZ157/CHN/2021 all contain eight genes in the order of 3'-N-P/V/C-M-F-SH-TM-G-L-5'. Moreover, HBJZ157/CHN/2021 and BeiV contain a putative ORF-X, the occurrence and function of which are currently unknown. Additionally, HBJZ120/CHN/2021 lacked the SH gene, a feature shared by Mount Mabu Lophuromys paramyxovirus 1 (MMLPV1), Mount Mabu Lophuromys paramyxovirus 2 (MMLPV2), Paju Apodemus paramyxovirus 1 (PAPV1), Ruloma virus, and other jeilongviruses. A schematic organization of the genomes of the two novel jeilongvirus-related strains with typical species (overview in Table S4) in the family *Paramyxoviridae* is shown in Fig. 2. The genome structures of the novel strains intriguingly resembled the genomes of other known representatives of the genus *Jeilongvirus*.

**Fig. 3:**
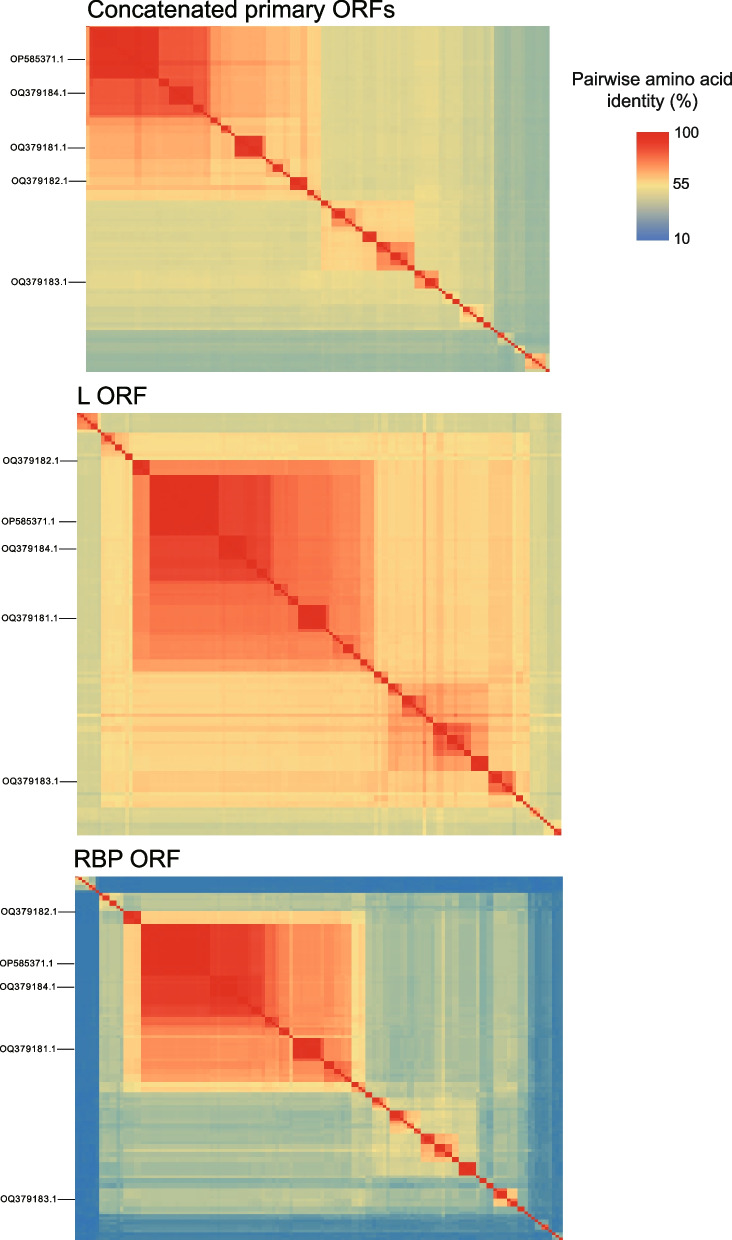
Pairwise amino acid identity matrices of novel strains (marked on the left of each matrix) with all jeilongviruses currently submitted and annotated in NCBI Virus. The matrices are detailed in Fig. S1, and virus and strain names are listed in Table S4. Three matrices were performed based on concatenated primary ORFs, L and RBP ORFs, respectively. Three heat maps were visualized in accordance with the scale in the top right

As shown in Fig. 3 and Figure S1, the following features can be revealed based on the identity matrices at the amino acid level: 1) Throughout the coding regions in species from the genus *Jeilongvirus* and jeilongvirus-related strains, the N, M, F, and L ORFs were relatively conserved, whereas lower identities were found in the P and receptor binding protein (RBP) ORFs. 2) Regarding the overall identity of genomes (excluding untranslated regions) from diverse viruses based on BioAider v1.423, HBJZ157/CHN/2021 shared the highest identity (99.28%) at the amino acid level with Rattus tanezumi Jeilongvirus RT-K-07. Similarly, HBJZ120/CHN/2021 was the most similar to PAPV-2, sharing 98.5% identity.

### Phylogenetic analysis of jeilongviruses

**Fig. 4:**
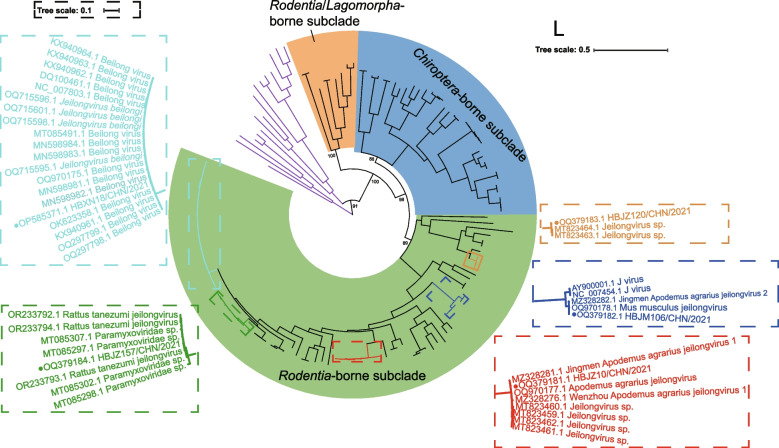
ML phylogenetic tree of jeilongviruses. The phylogenies were reconstructed by ML and based on amino acid of L protein. Viruses discovered in this study are marked with solid circles and bounded by larger dashed boxes with those are closely clustered (HBXN18/CHN/2021 cluster shown in cyan, HBJZ10/CHN/2021 cluster in red, HBJM106/CHN/2021 cluster in blue, HBJZ120/CHN/2021 cluster in gold, and HBJZ157/CHN/2021 cluster in green). The smaller adjacent dashed box in the same color shows the position in the tree. The outgroup is shown in purple. The five strains in the study were discovered in rodent samples (for instance, *Apodemus agrarius*, *Rattus norvegicus*) collected from Hubei Province in central China. According to the hosts and topology, strains in genus *Jeilongvirus* were classified into 3 subclades: *Rodentia*-borne subclade (covered in the green color), *Chiroptera*-borne subclade (covered in blue), and *Rodentia*/*Lagomorpha*-borne subclade (covered in orange). The ML tree was visualized in accordance with the scale in the top right and the magnified scale bounded by dashed boxes in the top left. The scale bars indicate 0.5 or 0.1 amino acid substitutions per site. Bootstrap values are shown for critical nodes

Three of the five representative jeilongvirus-related strains identified in this study were significantly similar to BeiV, JMAaJV-1, and JMAaJV-2. The phylogenetic analysis shown in Fig. 4 revealed that these strains clustered closely with those sharing the highest similarity, indicating that they could be recognized as viral species. The coding region of the L gene, located at the 5' terminus of the paramyxovirus genome, produces the L protein, which plays diverse roles as a conserved catalytic agent in RNA synthesis and processing [41–43]. Phylogenetic analysis based on amino acid sequences of L protein (Fig. 4) indicated that HBJZ120/CHN/2021 clusters with PAPV-2. HBJZ157/CHN/2021 clusters closely with Rattus tanezumi Jeilongvirus, Paramyxoviridae sp. RtBi-ParaV/Tt2013, and RtRt-ParaV/Tb2018. Moreover, strains in the genus *Jeilongvirus* can be classified into three subclades: *Rodentia*-borne subclade (carried by rodents), *Chiroptera*-borne subclade (carried by bats), and *Rodentia*/*Lagomorpha*-borne subclade (carried by rodents or *Ochotona cansus*) on the basis of topology in phylogeny and hosts (Fig. 4 and S2).

### Prevalence of jeilongviruses in rodents

To systematically investigate the prevalence of previously unexplored jeilongviruses in Hubei rodents (Fig. 1), we performed multiple RT-PCR or NPCR assays. In total, 71 samples were positive for BeiV (overall positivity rate: 6.38%) and were primarily distributed in Shiyan (17.92%), Xianning (5.26%), Xiangyang (7%), and Jingmen (4.27%). Only a few samples from Jingzhou (1.49%) were BeiV-positive. Details about the positive samples distributed among cities or hosts are shown in Table 2, from which it can be discovered that BeiV was primarily carried by *R. norvegicus* and *R. tanezumi*. BeiV was also found in *Apodemus agrarius* and *Niviventer confucianus*. In samples from Jingzhou, 24 of 202 rodents were JMAaJV-1-positive, and 14 of 202 were HBJZ120/CHN/2021-positive. Both viruses were found in *A. agrarius* (Table 2). JMAaJV-2-positive was detected in 4 of 202 rodents from Jingzhou and 15 of 164 rodents from Jingmen, mainly in *Mus musculus* (Table 2). HBJZ157/CHN/2021 was detected in three individuals of *R. tanezumi* from Jingzhou, China. Although no jeilongvirus coinfections were discovered, the distribution of jeilongviruses was found to be widespread in diverse regions and hosts.

**Table 2:** Prevalence of jeilongviruses in rodents collected from Hubei Province, China

No. of positive samples/ no. of detected samples (Per. positivity)											
**Virus names**		**Cities**	**Host species**								
			*Rattus norvegicus*	*Rattus tanezumi*	*Leopoldamys edwardsi*	*Rattus lossea Swinhoe*	*Rattus nitidus Hodgson*	*Mus musculus*	*Apodemus agrarius*	*Niviventer confucianus*	Total
Beilong virus		*Xianning*	9/88	0/33	0/2	0/3	0/1	0/1	0/27	0/16	9/171 (5.26%)
		*Shiyan*	16/71	17/68	━	━	━	0/64	2/5	3/4	38/212(17.92%)
		*Xiangyang*	14/90	0/52	━	━	━	0/13	0/45	━	14/200(7%)
		*Jingmen*	7/35	0/27	━	0/6	━	0/92	━	0/4	7/164(4.27%)
		*Jingzhou*	3/7	0/46	━	━	━	0/23	0/126	━	3/202(1.49%)
		Other cities	0/62	0/40	━	━	━	0/23	0/39	━	0/164(0%)
		Total	49/353(13.88%)	17/266(6.39%)	0/2(0%)	0/9(0%)	0/1(0%)	0/216(0%)	2/242(0.83%)	3/24(12.5%)	**71/1113(6.38%)**
Jingmen Apodemus agrarius jeilongvirus 1		*Jingzhou*	0/7	0/46	━	━	━	0/23	24/126	━	24/202(11.88%)
		*Jingmen*	0/35	0/27	━	0/6	━	0/92	━	0/4	0/164(0%)
Jingmen Apodemus agrarius jeilongvirus 2		*Jingmen*	0/35	0/27	━	0/6	━	15/92	━	0/4	15/164(9.15%)
		*Jingzhou*	0/7	0/46	━	━	━	3/23	1/126	━	4/202(1.98%)
HBJZ120/CHN/2021		*Jingzhou*	0/7	0/46	━	━	━	0/23	14/126	━	14/202(6.93%)
		*Jingmen*	0/35	0/27	━	0/6	━	0/92	━	0/4	0/164(0%)
HBJZ157/CHN/2021		*Jingzhou*	0/7	3/46	━	━	━	0/23	0/126	━	3/202(1.49%)

## Discussion

Many similarities in genomic organization were observed between the novel strains (HBJZ120/CHN/2021 and HBJZ157/CHN/2021) discovered in this study and established jeilongvirus taxa, adhering to the genome structure 3′-N-P/V/C-M-F(–SH)–TM-G-L-5′, carrying conserved sequences of gene start, stop, and IGR. Moreover, analysis of pairwise amino acid sequence identities and phylogenetic trees based on the L protein or concatenated primary ORFs revealed that HBJZ120/CHN/2021 closely clustered with PAPV-2 in the genus *Jeilongvirus*, whereas HBJZ157/CHN/2021, RtRt-ParaV/Tb2018, and RtBi-ParaV/Tt2013 closely clustered within the genus *Jeilongvirus*. According to the jeilongvirus species distinctive criterion [13, 44], which is based on a distance > 0.03 using the complete L protein, it was further confirmed that HBJZ120/CHN/2021 and HBJZ157/CHN/2021 are not separate species but novel strains belonging to the genus *Jeilongvirus*.

Rodent specimens were collected from seven cities in Hubei Province across various natural habitats such as forests, caves, and swamps, as well as artificial habitats such as agricultural, residential, and industrial areas. These sampling sites are located in a subtropical monsoon climate region and represent typical ecological conditions in central China. Analyzing HTS raw data indicated that BeiV presented a broad distribution, whereas the other four viruses appeared locally. NPCR and multiple RT-PCR were used to investigate the prevalence of jeilongviruses. Given that HBJZ157/CHN/2021 was present at a low abundance, NPCR was preferred. Multiple RT-PCR was used to detect multiple viral genes (*e.g.* P, G, and L genes of BeiV), and a positive result was defined as the occurrence of multiple strips, inevitably leading to lower positive percentages compared to conventional RT-PCR. Furthermore, experimental confirmation of the pooled primer sets is required to ensure specificity and absence of interference. Nevertheless, multiple RT-PCR can more effectively reflect the integrity of viral genomes in samples and reduce the likelihood of misinterpreting gene fragment contamination as viral presence. The prevalence of jeilongviruses can support diverse analyses; for example, the overall positive percentage of BeiV in the investigation was 6.38% (71/1113), which provides data from central China and improves the multiple logistic regression analysis by Chen et al. [32]. As shown in Table S5, the ranges of positive sites and rodent hosts of *Jeilongvirus beilongi* and *Jeilongvirus apodemi* were relatively extensive, while those of other jeilongviruses were much narrower, possibly due to a relatively poor understanding of these viruses or a lack of sufficient epidemiological research. From our findings, we conclude that jeilongviruses are widespread among wild rodents.

Unfortunately, this study did not include serological investigations of rodents or susceptible populations in viral hotspots. Considering the difficulty of trapping live rodents, we will focus on sentinel hospitals and collecting serum samples from patients to further explore the prevalence of jeilongviruses in populations. Although we did not isolate novel strains in this study, jeilongvirus strains should be subject to further investigation to better elucidate their pathogenicity and infectivity.

## Conclusions

In this study, we collected 1,113 rodents from Hubei Province in China and divided the specimens into 29 pools based on their tissues, regions of origin, and species. We assembled and annotated 30 jeilongvirus-related contigs using HTS and a virome workflow. After correcting the genomes and analyzing the genome structure and phylogeny based on the L protein, we identified five separate strains among the nearly complete genomes, including two novel strains (HBJZ120/CHN/2021 and HBJZ157/CHN/2021) and three previously discovered viruses (BeiV, JMAaJV-1, and JMAaJV-2). Jeilongviruses were categorized into three subclades based on their phylogenies and hosts. Furthermore, we determined the prevalence of latent jeilongviruses in rodents from Hubei through PCR screening and found that the distribution of jeilongviruses was widespread, with BeiV exhibiting the greatest abundance across studied regions and host species.

### Supplementary Information

Supplementary Material 1.

Supplementary Material 2.

Supplementary Material 3.

Supplementary Material 4.

Supplementary Material 5.

Supplementary Material 6.

Supplementary Material 7.

## Data Availability

The genome sequences generated in this study are available in GenBank under accession numbers OQ379181-OQ379184 and OP585371.
