# Supplementary material for: Discovery and characterization of novel jeilongviruses in wild rodents from Hubei, China
Source: Virol J. 2024 Jun 25;21:146. doi: 10.1186/s12985-024-02417-8 (PMC11201313; doi:10.1186/s12985-024-02417-8)
Supplement: Supplementary file 6 — Supplementary Material 6. [file 12985_2024_2417_MOESM6_ESM.pdf]

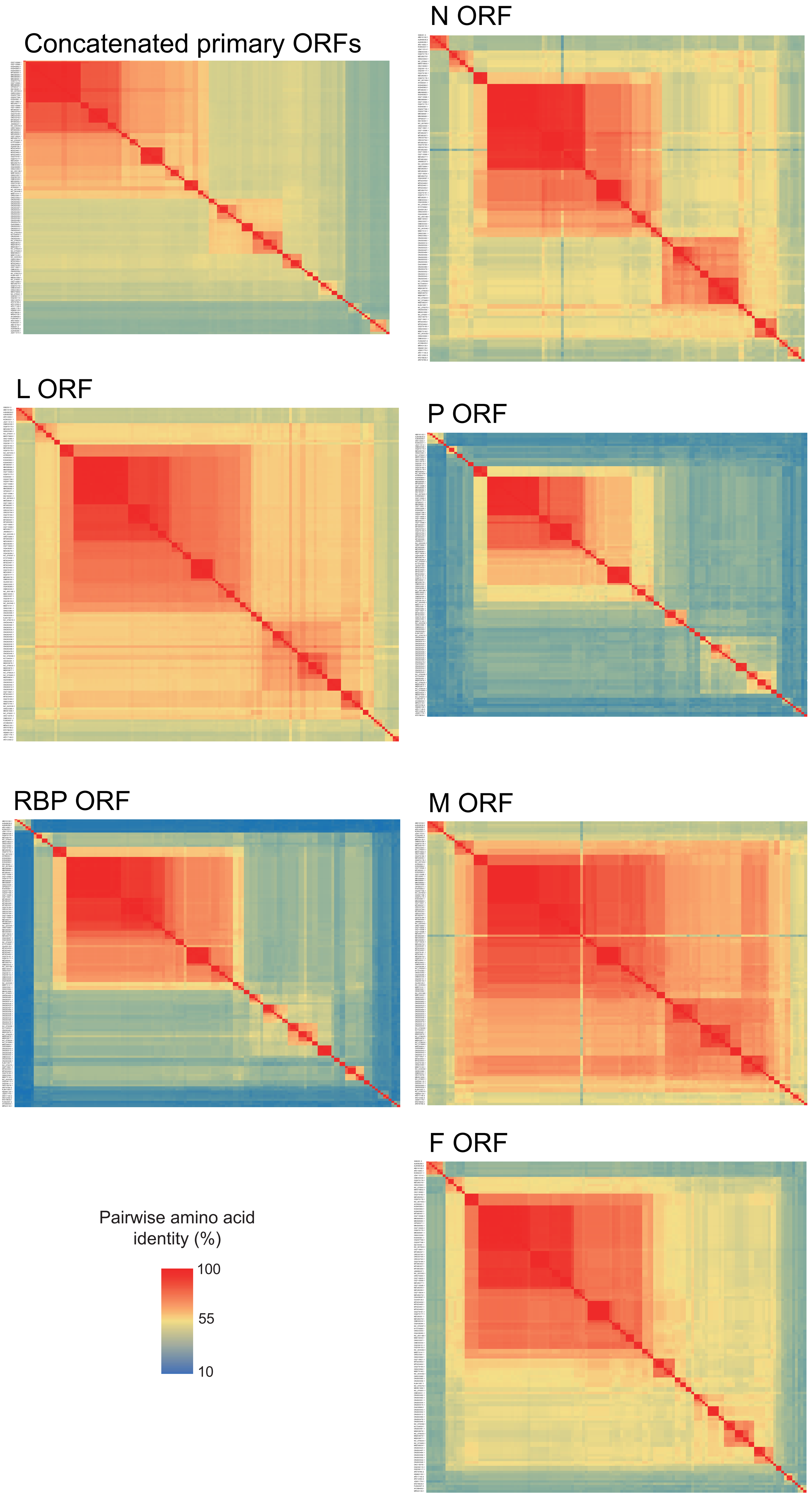

Fig. S1. Pairwise amino acid identity matrices of novel strains with all jeilongviruses (marked on the left of each matrix) currently submitted and annotated in NCBI Virus. The specific information of virus and strain names is listed in Table S4. Seven matrices were performed based on concatenated primary ORFs, N, L, P, RBP, M, and F ORFs respectively. Heat maps were visualized in accordance with the scale in the bottom left.
