# Supplementary material for: Discovery and characterization of novel jeilongviruses in wild rodents from Hubei, China
Source: Virol J. 2024 Jun 25;21:146. doi: 10.1186/s12985-024-02417-8 (PMC11201313; doi:10.1186/s12985-024-02417-8)
Supplement: Supplementary file 7 — Supplementary Material 7. [file 12985_2024_2417_MOESM7_ESM.pdf]

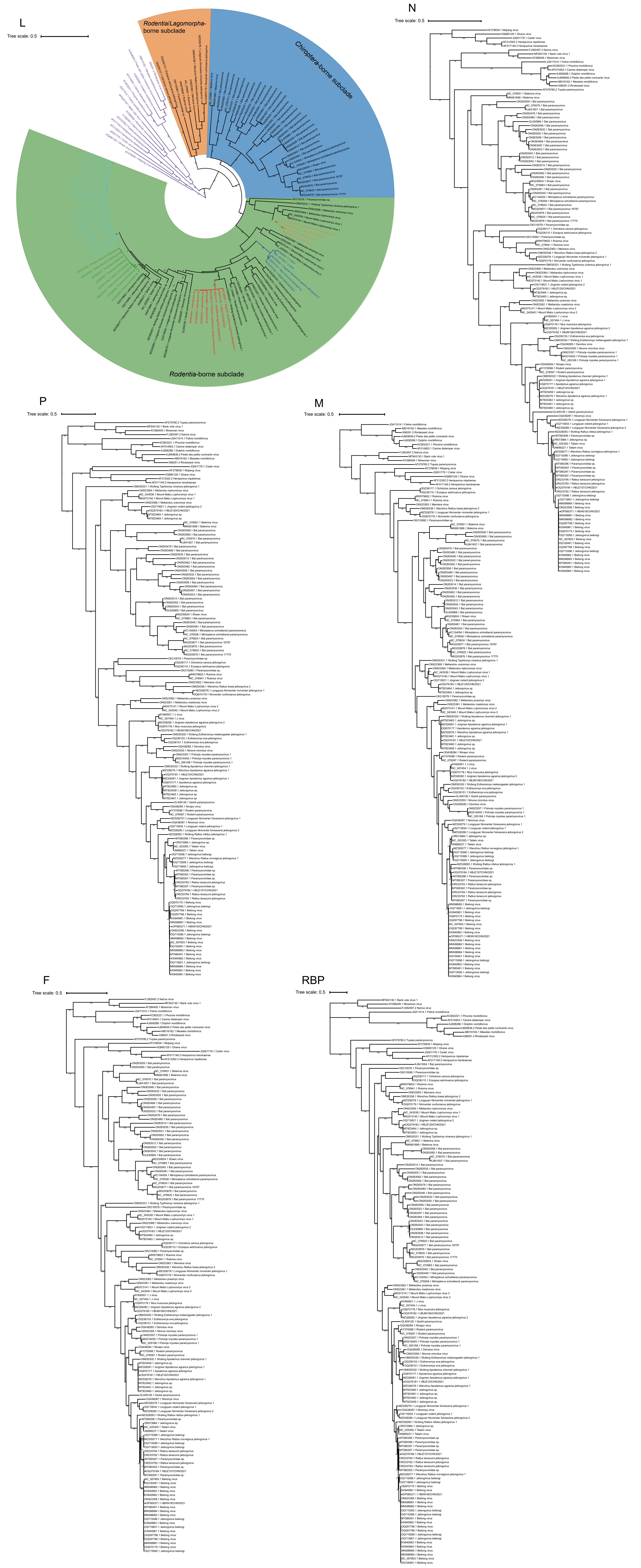

Fig. S2. ML phylogenetic trees of jeilongviruses. The phylogenies were reconstructed by ML method and based on amino acid sequences of L, N, P, M, F, and RBP proteins. Viruses discovered here are marked with solid circles. The outgroup is colored by purple in the phylogeny of L protein. According to the hosts and topology, strains in genus *Jeilongvirus* are classified into 3 subclades: *Rodentia*-borne subclade (covered in the green color), *Chiroptera*-borne subclade (covered in blue), and *Rodentia*/*Lagomorpha*-borne subclade (covered in orange). The ML trees were visualized in accordance with the scale in the top left of each tree. The scale bars indicate 0.5 amino acid substitutions per site and bootstrap values are shown for major nodes.
